# Unmanned Remotely Operated Search and Rescue Ships in the Canadian Arctic: Exploring the Opportunities, Risk Dimensions and Governance Implications

**DOI:** 10.1007/978-3-030-44975-9_5

**Published:** 2020-03-17

**Authors:** Jinho Yoo, Floris Goerlandt, Aldo Chircop

**Affiliations:** 2 Marine & Environmental Law Institute, Schulich School of Law, Dalhousie University, Halifax, NS Canada; 3 Department of Industrial Engineering, Dalhousie University, Halifax, NS Canada; 4 Marine Affairs Program, Dalhousie University, Halifax, NS Canada; 5 Department of Industrial Engineering, Dalhousie University, Halifax, NS Canada; 6 Marine & Environmental Law Institute, Schulich School of Law, Dalhousie University, Halifax, Canada; 7 Department of Industrial Engineering, Dalhousie University, Halifax, NS Canada

**Keywords:** Canadian Arctic, Governance, Risk management, Risk prevention measures, Remotely operated search and rescue ships, Unmanned ships

## Abstract

This chapter is a proactive risk exploration of hypothetical remotely operated search and rescue (SAR) ships in the Canadian Arctic. The harsh and remote environment in the region, combined with complicated coastlines and many uncharted or poorly charted traffic routes, makes it one of the most challenging SAR areas. Canada has committed itself to safety, environmental protection and sovereign presence in the area by maintaining joint SAR centres of federal government departments and mobilizing private volunteers. The characteristics of Canadian SAR response in the Arctic rest with its high dependency on heavy equipment such as aircraft, helicopters and icebreakers, entailing prolonged hours of response time. As recent climate change impacts and maritime traffic increase in the northern waters disclose safety gaps, innovation in SAR assets is anticipated. The safety gaps may be filled by state-of-the-art remote control technology. This chapter discusses remotely operated unmanned ships for SAR response, exploring their opportunities, risk dimensions and governance implications.

## Introduction

Could we imagine a ship remotely controlled from a distance of 8000 km? This long-distance test has been successfully passed, and a vessel-borne sensor with machine learning has advanced to identify the brand name of beer cans in the water (Wärtsilä 2017; Baraniuk 2017). The *Yara Birkeland*, an 80 m-long autonomous cargo ship, is expected to be in service within a few years (Baraniuk 2017). Notably in May 2019, the first-ever remotely controlled cargo ship completed a 22 h-long voyage between the United Kingdom and Belgium with a maximum payload capacity of 2.5 tonnes (Amos 2019).

Drones have been tested in the Canadian Arctic for safety monitoring since the first remote trial at Alma, Quebec, in June 2017 (Transport Canada 2018). One Australian drone successfully searched and rescued persons at sea by dropping an inflatable life raft (Haddou-Riffi 2018). In line with these technical innovations, in May 2018, the International Maritime Organization (IMO) coined a new term, maritime autonomous surface ships (MASS), to describe these new technologies. MASS were categorized into four stages: (1) manual operation with automated processes and decision support, (2) manned remotely controlled ships, (3) unmanned remotely controlled ships and (4) fully autonomous ships (IMO 2018a).

Given the limitations of the present SAR response time (Chase 2013) and the safety risks to SAR responders in the Arctic, the authors anticipate the third stage of unmanned remotely controlled ships to be able to play a potential role as a breakthrough in SAR response. In particular, in parallel with the recent improvements of multitier communication systems, unmanned and remotely operated SAR ships (RO-SARS) could open a new phase of SAR operations, assisted by a tailored design for rescue operation in the Canadian Arctic. Considering wind, current and wave height effects, a preliminary conceptual design can feature a high-speed craft (HSC) of 24 m or more with a capacity of at least 12 passengers.

However, despite the probable benefits, unmanned RO-SARS would also face novel risks, raising safety concerns to various stakeholders (Aven and Renn 2010). This chapter aims at exploring the opportunities, risk dimensions and governance implications of unmanned RO-SARS in the Canadian Arctic context from the sociotechnical and legal perspectives. The discussion is guided by two research questions. First, given the Canadian northern SAR context, what opportunities and risk dimensions are anticipated if and when unmanned RO-SARS are deployed? Second, what governance implications and risk prevention measures can be drawn, considering preliminary risk assessment of RO-SARS under the International Risk Governance Council (IRGC) framework? The outcome of this exploratory analysis will likely contribute to developing a conceptual design, risk characterization and regulatory model of RO-SARS in later research.

## SAR in the Canadian Arctic Context

The International Convention on Maritime Search and Rescue, 1979 (SAR Convention), defines “search” as “an operation … to *locate* persons in distress” and “rescue” as “an operation to *retrieve* persons in distress, provide for their initial *medical* or other needs, and *deliver* them to a place of safety” (SAR Convention 1979, Annex, chap 1). These definitions are overarching principles in designing, manufacturing and operating RO-SARS.

### Navigational Complexity and Uncertainty in the Canadian Arctic

**Fig. 5.1:**
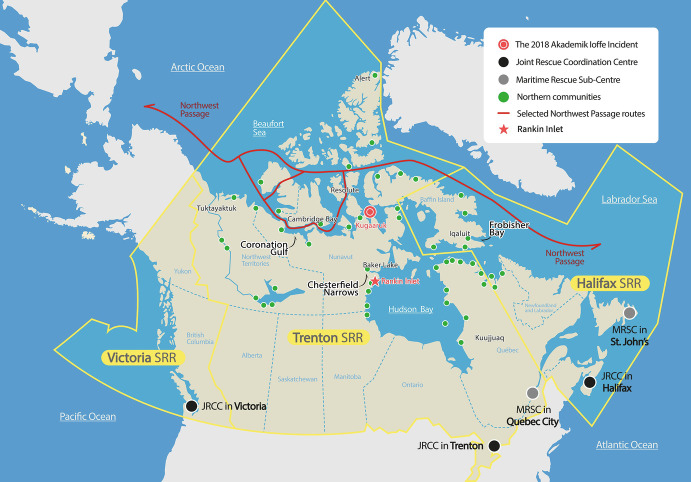
SAR centres in the Canadian Arctic and the 2018 *Akademik Ioffe* incident (Office of the Auditor General of Canada 2013, 2014)

The Canadian Arctic, and in particular the Northwest Passage (NWP), which extends 1450 km, is a uniquely complex navigational area consisting of multiple routes as shown in Fig. 5.1. Its characteristics include a combination of (1) a huge geographical area accounting for 40% of Canada’s land mass (Esri n.d.); (2) an extensive and complicated coastline with landfast ice in many areas; (3) an estimated 50,000 giant icebergs as well as drifting ice accompanied by strong winds, spray, fog and waves (Esri n.d.; Arctic Council 2009); (4) complicated sea routes through an archipelago consisting of over 90 major and 36,400 minor islands (World Atlas 2018); (5) the fact that only 10 per cent of the routes are considered adequately charted, although in some areas this figure has improved through recent surveys (Struzik 2018); (6) a complete lack of ports with any significant infrastructure; and (7) scarce emergency infrastructure for fuel, spare resources and trained personnel.

Most importantly, the Canadian Arctic is not only fundamental to Canada’s national identity but is the homeland of Indigenous peoples across the Yukon, the Northwest Territories and Nunavut. Canada has maritime boundary disputes and incomplete boundaries with the United States in the Beaufort Sea and Denmark in the Lincoln Sea (Government of Canada 2010). Most significantly, under international law, Canada claims a historic title to the waters of the Canadian Arctic archipelago over which it exercises sovereignty (Chircop et al. 2018). Shipping in the archipelagic waters and the territorial sea and the exclusive economic zone seaward of the straight baselines enclosing the archipelago is governed by stringent national law, most especially the Arctic Waters Pollution Prevention Act, 1970 (AWPPA), its regulations and the regulations under the Canada Shipping Act, 2001 (Fisheries and Oceans Canada 2009; Chircop et al. 2018).

### Multilevel Canadian SAR Resources in the Context of Increasing Demand

Canadian Arctic SAR is based on shared responsibilities of federal, territorial and municipal governments, as well as Indigenous communities, volunteers and commercial sectors (Senate of Canada 2018). SAR response entails reliance on heavy equipment, such as the dedicated 35 SAR aircraft (e.g., 17 fixed-wing and 18 rotary) operated by the Royal Canadian Air Force (2018), the 23 helicopters operated by the Canadian Coast Guard (2016) and the 15 icebreakers of the Canadian Coast Guard (2019) (Fisheries and Oceans Canada 2009; Senate of Canada 2018; Canadian Coast Guard 2019). These resources are mostly deployed in the three Joint Rescue Coordination Centres (JRCCs) and two Maritime Rescue Sub-Centres run by Canada’s Department of National Defence and the Canadian Coast Guard (CCG) (part of Fisheries and Oceans Canada) (Fig. 5.1). Volunteer SAR organizations include the Canadian Coast Guard Auxiliary with about 4000 volunteers and 1100 vessels across 16 bases in the Arctic; the Civil Air Search and Rescue Association (CASARA); and the Search and Rescue Volunteer Association of Canada (SARVAC) (Office of the Auditor General of Canada 2013). Commercial vessel operators, such as Fednav and Groupe Desgagnés, have also provided assistance.

Although these SAR resources appear to be considerable, the increased SAR demands in the northern region are presumed to exceed existing capabilities. The SAR resources are expected to cover both land and sea areas. Covering 18 million km^2^ of land and water, in 2017 the three JRCCs responded to about 10,000 air, marine and humanitarian incidents. Each JRCC addresses approximately 3000 incidents every year (Senate of Canada 2018; Office of the Auditor General of Canada 2013). Over 500 SAR missions were completed in the Canadian Arctic for the last 5 years immediately preceding 2019, compared with the yearly average of 29.3 accidents and incidents in the entire Arctic between 1995 and 2004 (Ward 2019; Arctic Council 2009).

In 2018, a CCG Arctic base was established in Rankin Inlet as part of Canada’s Arctic strategy to involve 14 northern Indigenous communities in SAR operations (Government of Canada 2009; Crown-Indigenous Relations and Northern Affairs Canada 2019). The Rankin Inlet Inshore Rescue Boat station in Nunavut will provide maritime SAR support during the summer season and will be crewed by Indigenous peoples trained by the Canadian Coast Guard (Canadian Coast Guard 2019).

## Opportunities of RO-SARS

### Increasing Vessel Traffic and Precursors of Arctic Accidents

The number of ship voyages to the Canadian Arctic increased from 123 in the year 2005 to 347 in 2016, including 147 voyages for cargo ships, 131 fishing vessels and 20 cruise/passenger ships (Lasserre 2018). Furthermore, there were 6036 cruise passengers in 2016, compared with 1239 in 2005 (Lasserre 2018). In 2017 alone, 178 vessels made about 400 visits to the Arctic including 32 transits through the NWP (LeBlanc 2018a).

Although there have not been massive fatalities in Northern Canada since the 1990s, some incidents could serve as precursors of disasters in the near future, such as the *Hanseatic* which ran aground with 149 passengers on board in 1996, the *Clipper Adventurer* which hit underwater ledges with 128 passengers in 2010 (TSBC 2012) and the *Akademik Ioffe* with 126 passengers, which was grounded in 2018 (Fig. 5.1) (TSBC 2019). It is plausible to assume that in case of more traffic entering the Arctic, the number of incidents will also increase. The year 2017 saw 71 marine incidents in the entire Arctic, up 29% year-on-year, with 29 total losses in the Russian Arctic and Bering Sea between 2008 and 2017 (Allianz 2018). Accordingly, it is reasonable to prepare for machinery damage and failure when navigating the Canadian Arctic, the conventional biggest cause of incidents in the region (Arctic Council 2009).

### Limitations of the Canadian Arctic SAR Response

Regardless of multilevel SAR resources, geographical remoteness and a complete lack of ports have created inherent limitations to the response time in the Canadian Arctic in the sense that how fast a response can be made depends on how close assets are located (Struzik 2018). Most importantly, the fact that all the JRCCs are located at the far south of the country (Fig. 5.1) has caused the average response time to be about 10 h under average ice conditions during the navigation season (Dalaklis 2019). In the *Akademik Ioffe* incident in 2018, the SAR flight took 9 h from the JRCC in Trenton, Ontario, to the grounding site (Fig. 5.1) (TSBC 2019; Struzik 2018). Similarly, SAR ships could take days to arrive at a site and rescue people (e.g., *Hanseatic* incident) due to the vast area and because the average speed of vessels on Arctic routes is known to be around 7 to 13 knots, compared with 21 to 25 knots in open sea (Plass et al. 2015). The number of people who can be delivered by helicopter is also extremely limited and helicopters need frequent refuelling stops.

Safety gaps and emerging risks have been mentioned at the federal level because of multifarious challenges: (1) the limited hydrographic survey and nautical charting of marine routes, (2) “dead zones” of radio communications, (3) the lack of trained SAR personnel, (4) ageing equipment, (5) insufficient icebreaking services, (6) the lack of land base connectivity through fibre optics, (7) the low bandwidth of satellite communications and (8) prolonged SAR time (Office of the Auditor General of Canada 2013, 2014; LeBlanc 2018a, b; Brown 2018). Given the financial burden of SAR amounting to over CAD 136.9 million (Canadian Coast Guard 2018), adding more aircraft, helicopters and icebreakers will not be a continuous and sustainable answer. Technical innovation in SAR is required.

### The Changing SAR Technology: Remote Control and Unmanned RO-SARS

#### Communication Links Under Innovative Improvement

**Fig. 5.2:**
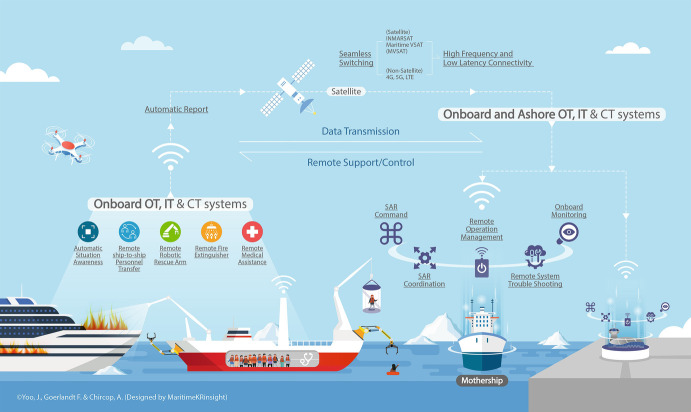
Preliminary system concepts of RO-SARS (the authors’ original concepts)

There have been multitier and hybrid approaches to improving marine communication technologies. Figure 5.2 provides a visual impression of some of these technologies: low earth orbit satellite services by 2022 (LeBlanc 2018b); Enhanced Satellite Communication Project, Polar (ESCP-P) (National Defence 2019); nano satellite and microsatellites called the “Gray Jay Pathfinder” (University of Toronto 2019; Boucher 2019; Cho 2019); and terrestrial systems by fibre-optic cables extending to the northern areas (Nuvitik Communications 2018). These developments are believed to gradually contribute to paving innovative foundations for effective SAR communication and response. For example, in May 2019, Canada opened the Marine Communication and Traffic Services Centre (MCTS) in Iqaluit, which provided assistance to 112 public and private vessels in the Northern Canada Vessel Traffic Services Zone (NORDREG) between 15 May and 31 July 2019 (Canadian Coast Guard 2019).

#### Remote Control Technology and Unmanned RO-SARS

Among enabling communication technologies, drones are surfacing as an effective tool to *search* in the Canadian Arctic, saving a significant share of the CAD 14,000 per hour needed for the operation of C130 Hercules aircraft. Drones may be equipped with thermal imaging devices, audio transmitter/receivers and emergency supplies payloads and are capable of streaming real-time images to a control centre (Ward 2019). In addition, unmanned RO-SARS could be a game changer in *rescue* operations, being equipped with a remote ship-to-ship personnel transfer crane, a robotic rescue arm to save people from the water, a remote fire extinguisher and a remote medical assistance service system.

On the deployment of RO-SARS in northern communities, JRCCs, and other stations, safety risks to SAR personnel would be eliminated; the response time would be accelerated; and cost-efficiency compared with icebreakers, aircraft and helicopters would be enhanced. Furthermore, a complementary solution would be provided to the otherwise insufficient SAR infrastructure, a lack of trained personnel, many uncharted areas and ageing equipment.

## Risk Dimensions of RO-SARS in the Canadian Arctic

### Pre-assessment of Risks

Given that technology is strongly associated with risk, it should be noted that a technology-led society could turn into a risk-susceptible society (United Nations 2017). Technology-driven risks could increase when *organized irresponsibility* begins to take advantage of unclear boundaries between ethics, law and technology (Beck 1999; FTI Consulting 2018). Modern risk society features the paradoxical coexistence of economic progress and increased risk, as well as unintended consequences and hidden risks between systems (Jarvis 2007; Renn et al. 2011). Accordingly, more attention should be paid to neighbouring risk components and an adaptive and integrative risk governance combining top-down and bottom-up approaches (Renn et al. 2011). In the maritime domain, risk is commonly defined as the probability of a defined hazard and the severity of its consequences (IMO 2018b). However, risk has been getting more complicated and uncertain in the maritime sector due to increasingly interconnected sociotechnical issues and related governance concerns. As such, the importance of explicitly and systematically considering uncertainties in the risk characterization has been stressed in recent years (Goerlandt and Reniers 2018).

As there are no unmanned RO-SARS in service in the Canadian Arctic at this time, the probability and severity of accidents are unknown. However, the expected minimum functional components could allow the pre-assessment of risks by exploring risk dimensions that could function as problem-framing, early warning and risk screening under the IRGC risk framework (Renn et al. 2011). This pre-assessment is based upon pre-existing maritime regulatory regimes, sociotechnical systems of ships and seafarers, the present stage of remote technology and the status of Canada’s SAR response. The functional heterogeneity of unmanned RO-SARS shown in Fig. 5.2 will likely define the nature of risks as systemic under the IRGC risk framework, meaning a risk of a high degree of complexity and uncertainty (Renn et al. 2011).

### Five Risk Dimensions to and from RO-SARS

#### Legality as a Threshold Issue

A potential concern with the deployment of RO-SARS vessels is their legal status. Canadian maritime law takes a broad definition of a ship in terms of navigability and a shipowner’s intent to use it as a ship (CSA 2001, s 2; Thibeault v. Canada 2015 FC 162). Unmanned and remotely operated submersibles have been considered as ships by Canadian courts (Cyber Sea Technologies Inc v. Underwater Harvester Remotely Operated Vehicle 2002 FCT 794). As such, it will not be difficult to have unmanned RO-SARS recognized as ships under Canadian maritime law with the usual consequences for safety, security, environment protection, insurability and liability. IMO has also defined MASS as a ship (IMO 2018b), and this characterization can thus be extended to unmanned RO-SARS. Again, the consequence of this definition is to bring MASS within the regulatory domains of international maritime safety, pollution prevention and security standards.

A further concern is whether the absence of a crew on board a SAR vessel might raise an issue of legality under the international law of the sea and international maritime law. The literature has explored possible solutions (Chircop 2017; Yoo and Shan 2019; IMO 2018c; Karlis 2018), which include amending regulatory provisions requiring seafarer presence on board, resorting to constructive treaty interpretation under the Vienna Convention on the Law of Treaties (VCLT 1969, art 61) or introducing new technology exemptions or equivalency under the International Convention for the Safety of Life at Sea (SOLAS) Convention (SOLAS 1974, regs I/4(b) and I/5). The effect of these solutions is to extend, to the extent appropriate, the full range of international rules and standards to MASS.

#### The Human Element in Seaworthiness

As in the case of all other ships, SAR vessels are required to be seaworthy. Pursuant to the definition of seaworthiness under the Canadian maritime law (Laing v. Boreal Pacific 2000 CanLII 16,313), RO-SARS as a ship should be reasonably fit in all respects, including SAR operations, to encounter the ordinary perils of the Canadian Arctic. “All respects” can be rephrased as the human and technical aspect of seaworthiness, which is a central principle in maritime law, to ensure the safety of ships under Article 94 of the United Nations Convention on the Law of the Sea (UNCLOS 1982).

The human element in seaworthiness emphasizes the role of a master and crew (SOLAS 1974, regs V/34–1; CSA 2001, s 109(1); Marine Personnel Regulations, s 215, 216). In particular, the International Convention on Standards of Training, Certification and Watchkeeping for Seafarers (STCW 1978, Annex, chaps II and III) sets mandatory minimum qualification standards for masters, officers and watch personnel. With respect to RO-SARS, the assets deployed will not be crewed but will be operated by an onshore team or operators on board motherships. SAR remote controllers will be required to exercise command skills and make tough decisions in a SAR value chain (Aase and Jabour 2015), but without having the benefit of at-sea situational awareness. Because irreparable consequences could occur by any failure of remote controllers on a real-time basis to understand, direct and cooperate with people and ships in distress and other SAR units, it is imperative that remote controllers are appropriately qualified according to generally accepted training standards (Schmied et al. 2017). Among other things, remote controllers will have to be familiar with Volume III of the International Aeronautical and Maritime Search and Rescue Manual, 2000, as amended (IMO 2019). Moreover, remote controllers should be conversant with the rules of the road and ice navigation because 70% of navigational negligence worldwide is attributable to the violation of the rules of steering and sailing (Maritime News 2019). Indeed, remote technology and the human element are inseparable (Rothblum 2000), and it will be necessary to establish uniform standards for SAR remote controllers and simulator-based remote SAR training programs.

#### The Technical Element of Seaworthiness

Seaworthiness of unmanned RO-SARS should be verified for reliability of interconnectivity and interoperability between operation technology (OT) (e.g., sensors and software of situation awareness), information technology (IT) (e.g., data collection, storage and analysis) and communication technology (CT) (e.g., satellite and terrestrial communication systems) in Canadian Arctic operations. Any failure of these technical components of RO-SARS could cause serious and unanticipated hazards to the safety of the SAR operation. In addition to hull structure, engines, machinery, electrical systems and conventional ship equipment, special attention will need to be paid to remote technical elements, including sensor technology, communication links, cyber safety and cybersecurity, and the interface between RO-SARS and remote controllers. Each of these four elements is discussed immediately below. More significantly, practical rescue functionality should be added to the conceptual design of RO-SARS. After all, human and technical elements of RO-SARS will have to prove that their reliability is as capable as the ordinary practice of conventional maritime rescue responders in the Arctic (c.f., Yoo et al. 2019).

A sensor is a device that responds to biological, chemical or physical stimulus such as heat, light, sound and pressure, providing a measured response of the observed stimulus (ISO 2011). Sensor function is critical for situation awareness in a SAR operation. It is enabled by the collection and integration of information from on-board sensors (e.g., heat, sonar and sound detection sensors), cameras and the automatic identification system (AIS) through satellites (Perera and Murray 2019). Even the lookout requirement under the International Regulations for Preventing Collisions at Sea (COLREG) (COLREG 1972, rule 5) is expected to be fulfilled by sensor technology (Lloyd’s Register 2017 Code, chap 10.1007/978-3-030-44975-9_4, s 4.1.5; Bruhn et al. 2014). However, the quality of data collected through sensors could be compromised by fog, rain, temperature, wind, freezing or harsh weather, which makes the resilience of sensor functions important when breakdowns occur (Bruhn et al. 2014; Lim 2019). As such, a system with a fail-safe design or sensor fusion has been suggested (Kim 2017).

The very recent development of communications technology and government efforts to improve coverage are enablers for unmanned RO-SARS in Canada’s Arctic. Even if sensor technology functions well, the data created from the sensors must be seamlessly transmitted to remote controllers as pictured in Fig. 5.2. For this to function effectively, RO-SARS will likely rely on a combination of multitier and hybrid satellites and terrestrial communications for ship-to-ship and ship-to-ashore data exchange (Aase and Jabour 2015). This combination will allow remote controllers to perform remote SAR operations (Fig. 5.2).

In 2017 the ransomware called “NotPetya” attacked the Maersk shipping line’s central computer system, halting operations at 76 port terminals and costing the shipowner about USD 300 million (Thomson 2017). Cyber incidents can be defined as an occurrence that results in adverse consequences to the entire OT, IT and CT of a ship and its related systems. For example, a virus could corrupt chart data held in an electronic chart display and information system (i.e., cybersecurity), and software controlling engines may malfunction due to a lack of compatibility with upgraded software (i.e., cyber safety) (Jorgensen 2018). Vulnerability may exist in virtual reality bridges, remote control centres and other communication systems. Accordingly, there needs to be stringent testing and certification of system safety and security, access control, security control, penetration testing and adoption of best practices for the protection of OT, IT and CT systems (Woo and Kim 2018; Bureau Veritas 2017, s 1, ss 2.6.2). In addition, a system of attack-safety will be necessary (Kim 2017). Recently, the IMO amended the requirements for an approved safety management system under the International Safety Management (ISM) Code (IMO 1993) to take into account cyber risk management (IMO 2017).

The work scope of SAR remote controllers will not be simple but will be comprehensive so as to include controlling, navigating, monitoring, searching and rescuing. These multifarious functions will require an ergonomic design of the physical and psychological work environment in remote control centres ashore or in motherships. Most importantly, a ship safety management system for RO-SARS and personnel ashore should be put in place as enjoined under the ISM Code (SOLAS 1974, chap 10.1007/978-3-030-44975-9_9), because there should be a strong link between the hazards of the actual operations of RO-SARS and the specific design of the safety management system (Valdez Banda et al. 2019).

#### Interaction with Ships in Distress and Other SAR Units

One probable concern of the stakeholders would be the interaction among remote controllers, ships in distress and other SAR units. First, to increase communication links, remote controllers and remote control centres could be stationed in motherships, northern communities or JRCCs (Fig. 5.2). Second, the operation of RO-SARS should be coordinated on-scene to ensure the most effective results with other SAR units engaged (SAR Convention 1979, Annex, chap 4, art 4.7). Third, to maximize the SAR performance and minimize communication error, remote controllers should be better trained and an experienced SAR personnel. Finally, for safer interplay with ships in distress and other SAR units, adaptive dynamic positioning (Witkowska and Śmierzchalski 2018), safe routeing systems (Lehtola et al. 2019), collision avoidance systems (Ozturk and Cicek 2019) and cooperative control algorithms (Almeida et al. 2010) are expected to be useful to RO-SARS.

#### Effective Design of RO-SARS

Finally, it is the design of unmanned RO-SARS that enables the rescue of people from waves, freezing temperatures and floating ice. At the design stage, depending on the nature of the voyages, the preliminary length of 24 m or more may entail meeting the requirements of the International Convention on Load Lines (ICLL 1966, Annex A, art 5). Further, having the capacity to carry more than 12 passengers will require RO-SARS of 15 tonnage or less to hold a “passenger vessel safety certificate” issued under the Vessel Certificates Regulations (2007, ss 3, 9, 10). As SOLAS defines a passenger ship to be a vessel carrying more than 12 passengers (SOLAS 1974, regs I/2 and II-1/1), the design of RO-SARS for an international voyage will have to factor in the requirements of SOLAS. Most importantly, an effective feasibility study on design should be made with respect to a ship-to-ship personnel transfer cranes, a robotic rescue arm to deliver people out of water, remote fire extinguishers and remote medical assistance. The authors of this chapter presume that unmanned RO-SARS could contribute to SAR response more likely with respect to *rescue* operations in coordination with other search equipment such as drones, satellites and aircraft. A further risk assessment of the conceptual design of RO-SARS should be made considering rescue-focused functionality, proper power systems for fast navigation comparable to a high-speed craft, energy sources available in the northern communities, the proper size and length of the asset for delivery of more than 12 persons, a reversionary mode of partly autonomous operation in communication dead zones and structural strength resistant to floating ice and heavy winds (Lee 2018).

### Summary

Given the five risk dimensions of RO-SARS, most aspects of risk dimensions, except the risk of legality, are contingent upon sociotechnical developments and technical decisions. This complexity and uncertainty makes it difficult to characterize the risk of RO-SARS as being tolerable or not under the IRGC risk framework. Regardless, this problem-framing could at least serve as an early warning and as a basis for specifying design requirements. Moreover, the advantages of RO-SARS, especially in actual rescue operations, will not be easily outweighed by these risk dimensions. Adaptive designs of unmanned RO-SARS and standardization of operation will likely serve as an innovative solution to lagging SAR response time.

## Governance Implications

Given the complexity and uncertainty of unmanned RO-SARS in the Canadian Arctic context, this risk-reducing SAR response mechanism necessitates close collaboration between multilevel governance systems ranging from international regulatory bodies to national institutions, to Indigenous peoples and to private volunteers (Renn et al. 2011). The human and technical risks of RO-SARS in Canadian Arctic waters will be controlled and managed by international and domestic regulatory regimes and include the engagement of Indigenous rights-holders and public and private stakeholders. In the near future, concern assessment and risk communication with rights-holders and stakeholders under the IRGC risk framework will be also needed.

### RO-SAR and International Conventions

Under Article 98 of UNCLOS, every state is obliged to require ships registered under its flag to render assistance to people and ships in danger, and every coastal state has a duty to promote the provision of infrastructure for adequate SAR services, emphasizing mutual regional arrangements between coastal states and neighbouring states. In the same vein, the SAR Convention also requires rescue coordination centres to be established by states (SAR Convention 1979, Annex, chap 10.1007/978-3-030-44975-9_2). Moreover, the Agreement on Cooperation on Aeronautical and Maritime Search and Rescue in the Arctic (Arctic SAR Agreement), to which Canada is a party, obligates states parties to implement the most expeditious border crossing procedures and establishes a legally binding duty of cooperation, including mutual SAR cooperation (Arctic Council 2011, arts 8, 9). Cooperation encompasses information exchange including available SAR facilities and lists of available supply infrastructure (Arctic Portal 2011). The Agreement is intended to enhance the cross-boundary mobility of SAR assets. Accordingly, the regional development and deployment of RO-SARS could be seen as supporting states parties’ duties under UNCLOS, the SAR Convention and the Arctic SAR Agreement.

### Multilayered Regulatory Regimes Applicable to RO-SARS

Besides the existence of an international SAR regulatory regime to which Canada is a party, RO-SARS deployed in and navigating the Canadian Arctic will also be governed by Canadian maritime law concerning registration, safety, security, environmental protection, insurability, tort and liability as they will likely be defined as a ship under Canadian maritime law. As “naval auxiliaries and other ships owned or operated by” government and “used only on government non-commercial service” are not bound by the safety of navigation regulations under SOLAS (SOLAS 1974, reg V/1), the safe navigation of RO-SARS owned or operated by the Canadian government will be mostly governed by Canadian national law. Moreover, Section 7 of the Canada Shipping Act, 2001, also implies that RO-SARS owned by the government can avoid the Act by resorting to other new regulations and provides that RO-SARS owned or operated by the Canadian Forces are outside of its application, as well as SOLAS and the Polar Code, which applies to ships certified under SOLAS. However, it should be noted that even state-owned RO-SARS will be subject to COLREG, which applies to all ships (COLREG 1972, rule 1). On the other hand, privately owned RO-SARS on international voyages carrying more than 12 passengers, which are more than 24 m in length, are subject to the construction, equipment and inspection requirements of SOLAS, as well as the watertight and stability requirements of ICLL (Canadian Supplement to the SOLAS Convention, s 2.1.1.1; ICLL 1966, Annex A, art 5).

### Political and Social License from the Arctic States and Northern Communities

SAR operations in the Canadian Arctic have a probability of crossing land borders and maritime boundaries with the United States and Denmark (Greenland). Even if the federal government approves the operation of RO-SARS, other Arctic states might not welcome the novel technology in waters under their sovereignty or jurisdiction for safety and security reasons (Lee et al. 2018). Accordingly, political and social license in and between neighbouring Arctic states, territories, Indigenous peoples and northern communities is important (van der Vegt 2018). Without their support, the deployment of both RO-SARS and remote control centres in the North could face obstacles. Furthermore, for the effective governance of RO-SARS in the Canadian Arctic, the coordination among federal government departments, JRCCs, volunteer groups, northern communities and neighbouring states will be essential to obtaining full support from aeronautical, maritime and ground SAR units.

## Risk Prevention Measures: Future Research Needs

Rapidly advancing remote technology for ships is poised to open new chapters for small cargo delivery (Amos 2019), oil spill response (Maritime Logistics 2019) and tugboat operations (Martine 2019), which also suggests new opportunities for SAR response in the Canadian Arctic. However, the following risk prevention measures which require further research and development are suggested because “risks are created and selected by human actors” (Renn et al. 2011).

First, a concept design for RO-SARS needs to be defined in terms of their size, length, structure, machinery and SAR functionality. Second, a more complete risk characterization should be made with regard to the technical design specification of RO-SARS, one which also accounts for stakeholder concerns and risk perceptions. Third, the Canadian government should develop uniform standards of qualification, training and certification for search and rescue remote controllers in Arctic waters (SQ-SARC) in the near future. Fourth, a prototype of RO-SARS should be repeatedly tested, inspected and surveyed through sea trials to verify their human and technical seaworthiness and effective interaction with ships in distress, other SAR units and relevant technologies, including drones. Fifth, remote controllers should be qualified, trained and licensed seafarers for SAR operation under new legal standards that would need to be developed, possibly by the IMO as well as under Canadian federal law. Sixth, stakeholders should increase multitier satellite and terrestrial supports and data transmitters so that the interconnectivity of OT, IT and CT can meet the ordinary practices of SAR responders in the Canadian Arctic. Seventh, a safety management system specifically for unmanned RO-SARS should be put in place with approved training simulators and mandatory procedures (Dasgupta 2017). Eighth, the contribution of northern communities to the practical operation of remote controllers and remote control centres is a key to the success in SAR response in the region (Ward 2019). As such, including these communities in the conception, planning and design of the centres, as well as the associated operating procedures, is highly recommended. Finally, knowledge-sharing and promotion of best practices of RO-SARS ought to be taken up by the Arctic Council through the Protection of the Arctic Marine Environment Working Group, perhaps through its Arctic Shipping Best Practice Information Forum, or the Emergency Prevention, Preparedness and Response Working Group or in collaboration with both Working Groups (Dalaklis 2019).

## Conclusion

Given the Canadian Arctic context, unmanned and remotely controlled ships could considerably enhance and complement Canadian SAR capabilities, particularly rescue operations, by reducing response time, infrastructural costs and life risks to responders. However, there are complex and uncertain risks that can be identified under the IRGC risk framework: the qualification and certification of remote controllers, the technical reliability of sensor technology, the stability of communication links, the hazards arising from the breach of cyber safety and cybersecurity, the probable interface errors between RO-SARS and remote controllers and the new design requirements for remote rescue functions such as the remotely operated ship-to-ship personnel transfer crane. These risks should be addressed under multilevel governance systems comprising international and national and public and private stakeholders. Most significantly, it is clear that unmanned RO-SARS are in line with the international conventions concerning SAR operations, that there are multilayered regulatory regimes applicable to these novel ships and that these vessels and craft should gain political and social license from the Arctic states and northern communities.

Nonetheless, the risks of deploying unmanned RO-SARS in the region should not be treated as simple. These risks feature a combination of intrinsic heterogeneities such as remote technology, the SAR operation itself and the extreme environment, all of which are complex and uncertain in nature under the terms of the IRGC risk framework. Indeed, although the opportunities for RO-SARS look promising, the required technical reliability and actual SAR practicability are unproven in the Arctic context. Hence, it is premature to characterize overall risks as intolerable, tolerable or acceptable. However, the minimal exploration of the risk dimensions taken in the foregoing discussion might trigger feasibility studies and dedicated ship design approaches accounting for the different hazards originating from this novel technology concept, for which new design approaches for unmanned vessels could be applied.
